# Cholinesterase is Associated With Prognosis and Response to Chemotherapy in Advanced Gastric Cancer

**DOI:** 10.3389/pore.2021.580800

**Published:** 2021-03-25

**Authors:** Yanzhi Bi, Junling Zhang, Dongxiang Zeng, Lili Chen, Wei Ye, Quanliang Yang, Yang Ling

**Affiliations:** ^1^ Department of Oncology, Changzhou Tumor Hospital Affiliated to Soochow University, Changzhou, China; ^2^ The Medical Department, 3D Medicines Inc., Shanghai, China; ^3^ Department of Hematology, The Suqian Affiliated Hospital of Xuzhou Medicine University, Suqian, China

**Keywords:** cholinesterase, prognosis, gastric cancer, chemotherapy, progression-free survival, overall survival

## Abstract

**Background:** Cholinesterase (CHE) is a routine serum biomarker in gastric cancer (GC). However, little research has been done on its clinical value in advanced GC. In addition, it is not clear whether it can be used as biomarker for the response and prognosis of advanced GC patients.

**Methods:** Between Jan. 2013 and Dec. 2016, a total of 150 patients with advanced GC treated with first-line chemotherapy were admitted to Changzhou Tumor Hospital Affiliated to Soochow University. We retrospectively identified serum CHE level on the day before chemotherapy and at the end of chemotherapy and abstracted clinicopathologic features and treatment outcomes. Univariate and multivariate survival analyses were performed to assess the relationship between serum CHE levels and progression-free survival (PFS) and overall survival (OS).

**Results:** A total of 150 advanced GC patients were included and divided into serum level ≥5,000 IU/L and serum level <5,000 IU/L. CHE level lower than 5,000 IU/L was associated with poorer PFS (HR, 1.60; 95% CI, 1.141–2.243; *p* = 0.006), poorer OS (HR, 1.76; 95% CI, 1.228–2.515; *p* = 0.002) and trend of poorer response (HR, 0.56; 95% CI, 0.272–1.129; *p* = 0.104). In univariate and multivariate logistic regression analysis, only liver metastasis and PS score were significantly associated with objective response (*p* < 0.05). The medium PFS was 8.0 months in patients with post-treatment CHE increased vs. 3.8 months in patients with CHE decreased after chemotherapy (HR, 1.82; 95% CI 1.28–2.57; *p* = 0.0002). The medium OS was 13.1 months in patients with increased post-treatment CHE vs. 8.1 months in patients with decreased post-treatment CHE (HR, 1.87; 95% CI 1.29–2.71; *p* = 0.0002).

**Conclusion:** Advanced GC with CHE levels below 5,000 IU/L was significantly associated with poor PFS and OS. The results suggested that CHE analysis before chemotherapy was a promising prognostic marker for advanced GC.

## Introduction

Gastric cancer (GC) is one of the most common malignancies [1]. The incidence and mortality of gastric cancer vary by region, but more than 50% cases occur in East Asia [2]. The GC was responsible for 10.6% of new cases and 13.6% of cancer-related deaths in China [3]. Due to the lack of typical symptoms of early gastric cancer, most patients with gastric cancer were diagnosed in the late stage of the disease with extensive regional lymph node involvement and/or invasion of adjacent structures. However, the prognosis for patients with advanced GC remains poor, with median survival times of 10–13 months [4]. It is recognized increasingly that it is not only the intrinsic properties of tumor cells that determine tumor spread but also the nutritional status of patients [5].

Cholinesterase (CHE) is a glycoprotein synthesized by the liver and secreted into the blood. It exists *in vivo* in the multiple forms of isozyme and is widely found in most tissues such as livers, kidneys and intestines [6]. Cholinesterase activity was reported to be decreased in heart attack, liver dysfunction, cancer metastasis, muscular tremors, and neurological disorders [7]. In addition, low serum CHE levels have been reported to be associated with the poor prognosis of many tumors, such as prostate cancer, uterine cervical cancer, pancreatic cancer [8–10].

Although CHE has been recognized as conventional serum biomarker in GC, little is known about the prognostic implications of CHE. In the current study, we investigated the clinicopathologic features and CHE and the impact on progression free survival (PFS) and overall survival (OS) following first line chemotherapy in advanced GC.

## Materials and Methods

### Study Design

We retrospectively reviewed advanced GC patients undergoing first line chemotherapy at Changzhou Tumor Hospital Affiliated to Soochow University between January 2013 and December 2016. All patients were administered chemotherapy as they were all at stage IV. Two patients were only treated with paclitaxel single-agent chemotherapy. Ninety-one patients received two-drug combination therapy including XELOX (capecitabine and oxaliplatin), TF (paclitaxel and 5-FU), SOX (S-1 and oxaliplatin), TS (paclitaxel + S-1), TP (paclitaxel and cisplatin) or FP chemotherapy (5-FU and cisplatin). Fifty-seven patients received three-drug combination traetment including DCF (docetaxel and cisplatin and 5-FU), ECF (epirubicin and cisplatin and 5-FU) or FOLFOX4 (Oxaliplatin and calcium folate and 5-FU). The following clinical characteristics before chemotherapy were abstracted: age, sex, CHE level, carbohydrate antigen 19-9 (CA19-9), carcinoembryonic antigen (CEA), an Eastern Cooperative Oncology Group (ECOG) performance status (PS), tumor differentiation degree, histological type, tumor location, surgery, response to first line chemotherapy, PFS, and OS. On the day before the first chemotherapy cycle (pre-chemotherapy) and the last day of the final chemotherapy cycle (post-chemotherapy), serum was collected and analyzed for the level of CHE. Patients were divided into two groups, serum level ≥5000 IU/L and serum level <5,000 IU/L.

The study was approved by relevant regulatory and independent ethics committee of the Changzhou Tumor Hospital Affiliated to Soochow University and done in accordance with the Declaration of Helsinki and the International Conference on Harmonization Good Clinical Practice guidelines.

### Statistical Analyses

Progression free survival (PFS) was defined as the time from the date of first line chemotherapy administration to the progression of cancer, or death from any cause. Overall survival (OS) was calculated from the date of first line chemotherapy administration to the date of death from any cause. Survival description was illustrated by the Kaplan-Meier curves, with *p* value determined by a log-rank test. Hazard's ratio (HR) was determined through the univariate and multivariate Cox regression. The associations between response and variables were examined by a univariate logistic regression. Variables with significant *p* values or interest were included into multivariate logistic regression. Continuous variables were compared by Mann-Whitney *U* test. For all analyses, *p* value <0.05 was considered to be statistically significant, and a CI of 95% was used (95% CI). All statistical analyses were performed using SPSS22.0 software (SPSS, Inc., Chicago, IL, United States).

## Results

### Patient Characteristics

One fifty patients with advanced GC treated with first-line chemotherapy were included in this analysis. The baseline characteristics were summarized in Table 1. 111 of 150 patients (74.0%) were male and 51.3% patients were more than 65-years- old. Most patients had no liver metastasis (65.3%) or peritoneal metastasis (89.3%), respectively. Ninety-three patients (62.0%) received less than three drugs. The main tumor location of patients were cardia and/or gastric fundus (44.7%), body (30.7%) and antrum (23.3%). Only two patients were HER-2 positive which was lower than the reported data. Patients were divided into two groups, serum level ≥5,000 IU/L and serum level <5,000 IU/L. And there was no significant difference in clinicopathologic characteristics between patients with serum level ≥5,000 IU/ml and serum level <5,000 IU/L (Table 2).

**TABLE 1:** Baseline characteristics.

Variable	Total (n = 150)
Sex, n (%)	
Male	111 (74.0%)
Female	39 (26.0%)
Age (years)	
>65	67 (44.7%)
≤65	83 (55.3%)
Liver metastasis	
Yes	52 (34.7%)
No	98 (65.3%)
Peritoneal metastasis	
Yes	16 (10.7%)
No	134 (89.3%)
Chemotherapy regimen	
One and two drugs	96 (64.0%)
Three drugs	54 (36.0%)
CHE (IU/L)	
<5,000	57 (38.0%)
≥5,000	93 (62.0%)
CEA (ng/ml)	
<5	51 (34.0%)
≥5	99 (66.0%)
CA199 (U/ml)	
<37	79 (52.7%)
≥37	71 (47.3%)
ECOG performance status	
0–1	112 (74.7%)
>1	38 (25.3%)
Her-2 status	
Positive	2 (1.3%)
Negative	148 (98.7%)
Surgery	
Yes	68 (45.3%)
No	82 (54.7%)
Tumor differentiation	
High + medium	75 (50.0%)
Low	75 (50.0%)
Histological type	
Intestinal	50 (33.3%)
Diffuse	100 (66.7%)
Tumor location	
Body	45 (30.0%)
Antrum	35 (23.3%)
Cardia, gastric fundus	68 (45.3%)
Unknown	2 (1.3%)

CHE, cholinesterase; ECOG, Eastern Cooperative Oncology Group.

**TABLE 2:** Baseline clinicopathologic characteristics and pre-chemotherapy CHE Level.

Variable	CHE (IU/L)		
	<5,000 (n = 57)	≥5,000 (n = 93)	*p*
Sex, n (%)			
Male	40	71	0.446
Female	17	22	
Age (years)			
>65	28	39	0.403
≤65	29	54	
Liver metastasis			
Yes	25	27	0.078
No	32	66	
Peritoneal metastasis			
Yes	9	7	0.171
No	48	86	
Chemotherapy regimen			
One and two drugs	33	63	0.293
Three drugs	24	30	
CEA (ng/ml)			
<5	17	34	0.478
≥5	40	59	
CA199 (U/ml)			
<37	30	49	1.000
≥37	27	44	
ECOG performance status			
0–1	40	72	0.339
>1	17	21	
Her-2 status			
Positive	1	1	1.000
Negative	56	92	
Surgery			
Yes	21	47	0.129
No	36	46	
Tumor differentiation			
High + medium	29	46	1.000
Low	28	47	
Histological type			
Intestinal	19	31	1.000
Diffuse	38	62	
Tumor location			
Body	18	27	0.704
Antrum	14	21	
Cardia, gastric fundus	25	43	
Unknown	0	2	

### Association Between Pre-Chemotherapy CHE and Survival Outcomes

**FIGURE 1:**
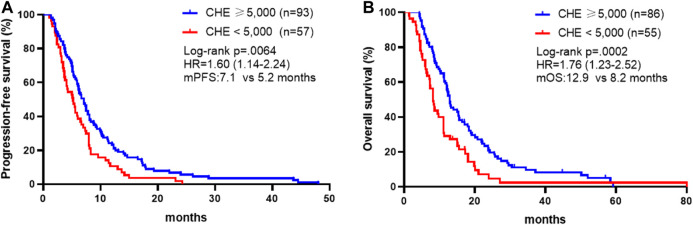
Association between CHE status and survival outcomes. **(A)** Kaplan-Meier survival curves of progression-free survival comparing patients with CHE ≥5,000 and patients with CHE <5,000. **(B)** Kaplan-Meier survival curves of overall survival comparing patients with CHE ≥5,000 and patients with CHE <5,000.

We first analyzed whether pre-chemotherapy CHE was associated with the survival outcomes of chemotherapy in advanced GC. Patients with CHE levels above 5,000 IU/L obtained better PFS compared to patients with CHE levels below 5,000 IU/L (mPFS: 7.1 vs. 5.2 months, *p* = 0.0064, Figure 1A). In the present study, univariable analysis revealed significant association between poorer PFS and CHE level lower than 5,000 IU/L, PS > 1, high + medium tumor differentiation degree, or diffuse histological type, while no relation between PFS and chemotherapy regimen, liver/peritoneal metastasis, CA199 or CEA (Table 3). In a multivariate model, CHE, PS score, surgery, CEA and diffuse histological type independent prognostic indicators for PFS (Table 3, *p* < 0.05).

**TABLE 3:** Univariate and multivariate analyses of progression-free survival.

Parameter	Univariate analysis			Multivariate analysis		
	HR	95% CI	*p*	HR	95% CI	*p*
Sex	1.012	0.700–1.463	0.948			
Male vs. female						
Age	0.961	0.696–1.328	0.811			
>65 vs. ≤ 65						
Liver metastasis	1.189	0.844–1.675	0.323	1.234	0.843–1.809	0.280
Yes vs. No						
Peritoneal metastasis	1.677	0.994–2.830	0.053	1.473	0.852–2.546	0.165
Yes vs. No						
Chemotherapy regimen	0.803	0.572–1.127	0.204			
Three drugs vs. one and two drugs						
CHE (IU/L)	1.600	1.141–2.243	0.006	1.829	1.257–2.661	0.002
≥5,000 vs. < 5,000						
CEA (ng/ml)	1.408	0.998–1.986	0.051	1.492	1.036–2.149	0.031
≥5 vs. < 5						
CA199 (U/ml)	1.141	0.825–1.578	0.424			
≥37 vs. < 37						
ECOG performance status	1.562	1.076–2.267	0.019	1.531	1.019–2.302	0.040
>1 vs. 0–1						
Surgery	1.350	0.973–1.872	0.072	1.985	1.387–2.840	0.000
Yes vs. No						
Tumor differentiation	0.666	0.480–0.923	0.015	0.726	0.500–1.054	0.092
High + medium vs. low						
Histological type	1.844	1.296–2.624	0.001	1.738	1.142–2.644	0.010
Diffuse vs. intestinal						
Tumor location	1.021	0.718–1.452	0.907			
Body vs. antrum						

CHE, cholinesterase; ECOG, Eastern Cooperative Oncology Group.

In consistence with PFS, better OS was discovered in patients with CHE levels above 5,000 IU/L compared to patients with CHE levels below 5,000 IU/L (mOS: 12.9 vs. 8.2 months, *p* = 0.0002, Figure 1B). Univariate and multivariate analysis exposed significant association between OS and CHE, peritoneal metastasis, PS score and tumor differentiation degree (Table 4, *p* < 0.05).

**TABLE 4:** Univariate and multivariate analyses of overall survival.

Parameter	Univariate analysis			Multivariate analysis		
	HR	95% CI	*p*	HR	95% CI	*p*
Sex	1.191	0.804–1.763	0.383			
Male vs. female						
Age	0.999	0.980–1.018	0.907			
>65 vs. ≤ 65						
Liver metastasis	1.574	1.086–2.279	0.016	1.494	0.983–2.271	0.060
Yes vs. No						
Peritoneal metastasis	2.433	1.428–4.145	0.001	2.034	1.170–3.536	0.012
Yes vs. No						
Chemotherapy regimen	0.970	0.682–1.382	0.867			
Three drugs vs. one and two drugs						
CHE (IU/L)	1.757	1.228–2.515	0.002	1.697	1.134–2.538	0.010
≥5,000 vs. < 5,000						
CEA (ng/ml)	1.346	0.938–1.931	0.107	1.232	0.846–1.793	0.276
≥5 vs. < 5						
CA199 (U/ml)	1.121	0.794–1.583	0.516			
≥37 vs. < 37						
ECOG performance status	2.214	1.496–3.277	0.000	2.282	1.487–3.504	0.000
>1 vs. 0–1						
Surgery	0.767	0.540–1.089	0.138	1.067	0.727–1.566	0.742
Yes vs. No						
Tumor differentiation	0.639	0.452–0.903	0.011	0.608	0.408–0.907	0.015
High + medium vs. low						
Histological type	1.588	1.098–2.298	0.014	1.246	0.813–1.911	0.313
Diffuse vs. Intestinal						
Tumor location	1.155	0.801–1.666	0.440			
Body vs. antrum						

CHE, cholinesterase; ECOG, Eastern Cooperative Oncology Group.

### Association Between Pre-Chemotherapy CHE and Response

The objective response rate was 25 or 11% in patients with CHE levels above 5,000 IU/L or patients with CHE levels below 5,000 IU/L respectively in Table 5. In univariate logistic regression, patients with CHE levels below 5,000 IU/L exhibited a trend of poorer objective response (HR, 0.555; 95% CI, 0.272–1.129; *p* = 0.104), whereas liver-metastasis and PS > 1 were associated with objective response with hazard ratios of 2.171 (95% CI, 1.082–4.356, *p* = 0.029) and 0.323 (95% CI, 0.131–0.797, *p* = 0.014), respectively (Table 6). However, in multivariate logistic regression including CHE level, liver and peritoneal metastasis, CEA, PS score, surgery, tumor differentiation and histological type, only PS score was significantly associated with objective response (Table 6, *p* < 0.05).

**TABLE 5:** Response to chemotherapy according to RECIST criteria.

Variable	CHE ≥5,000 IU/L (n = 93)	CHE <5,000 IU/L (n = 57)	*p*
Best overall response—no. (%)			
Complete response	1 (1.06)	0 (0)	0.1196
Partial response	37 (39.78)	16 (28.07)	
Stable disease	41 (44.08)	25 (43.85)	
Progressive disease	14 (15.05)	16 (28.07)	

CHE, cholinesterase.

**TABLE 6:** Univariate and multivariate analyses of response.

Parameter	Univariate analysis			Multivariate analysis		
	HR	95% CI	*p*	HR	95% CI	*p*
Sex	1.634	0.737–3.620	0.227			
Male vs. female						
Age	0.979	0.502–1.910	0.951			
>65 vs. ≤ 65						
Liver metastasis	2.171	1.082–4.356	0.029	2.144	0.981–4.684	0.056
Yes vs. No						
Peritoneal metastasis	0.371	0.101–1.366	0.136	0.523	0.130–2.100	0.361
Yes vs. No						
Chemotherapy regimen	1.962	0.984–3.914	0.056			
Three drugs vs. one and two drugs						
CHE (IU/L)	0.555	0.272–1.129	0.104	0.459	0.209–1.008	0.052
≥5,000 vs. < 5,000						
CEA (ng/ml)	1.385	0.676–2.839	0.373	1.175	0.535–2.579	0.688
≥5 vs. < 5						
CA199 (U/ml)	0.673	0.343–1.323	0.251			
≥37 vs. < 37						
ECOG performance status	0.323	0.131–0.797	0.014	0.316	0.119–0.842	0.021
>1 vs. 0–1						
Surgery	0.732	0.373–1.439	0.366	0.569	0.264–1.228	0.151
Yes vs. No						
Tumor differentiation	1.562	0.797–3.064	0.194	1.574	0.692–3.582	0.279
High + medium vs. low						
Histological type	0.608	0.302–1.223	0.163	1.054	0.448–2.478	0.904
Diffuse vs. Intestinal						
Tumor location	0.833	0.399–1.740	0.627			
Body vs. antrum						

CHE, cholinesterase; ECOG, Eastern Cooperative Oncology Group.

### Association Between CHE Status Change After Chemotherapy and Survival Outcomes

**FIGURE 2:**
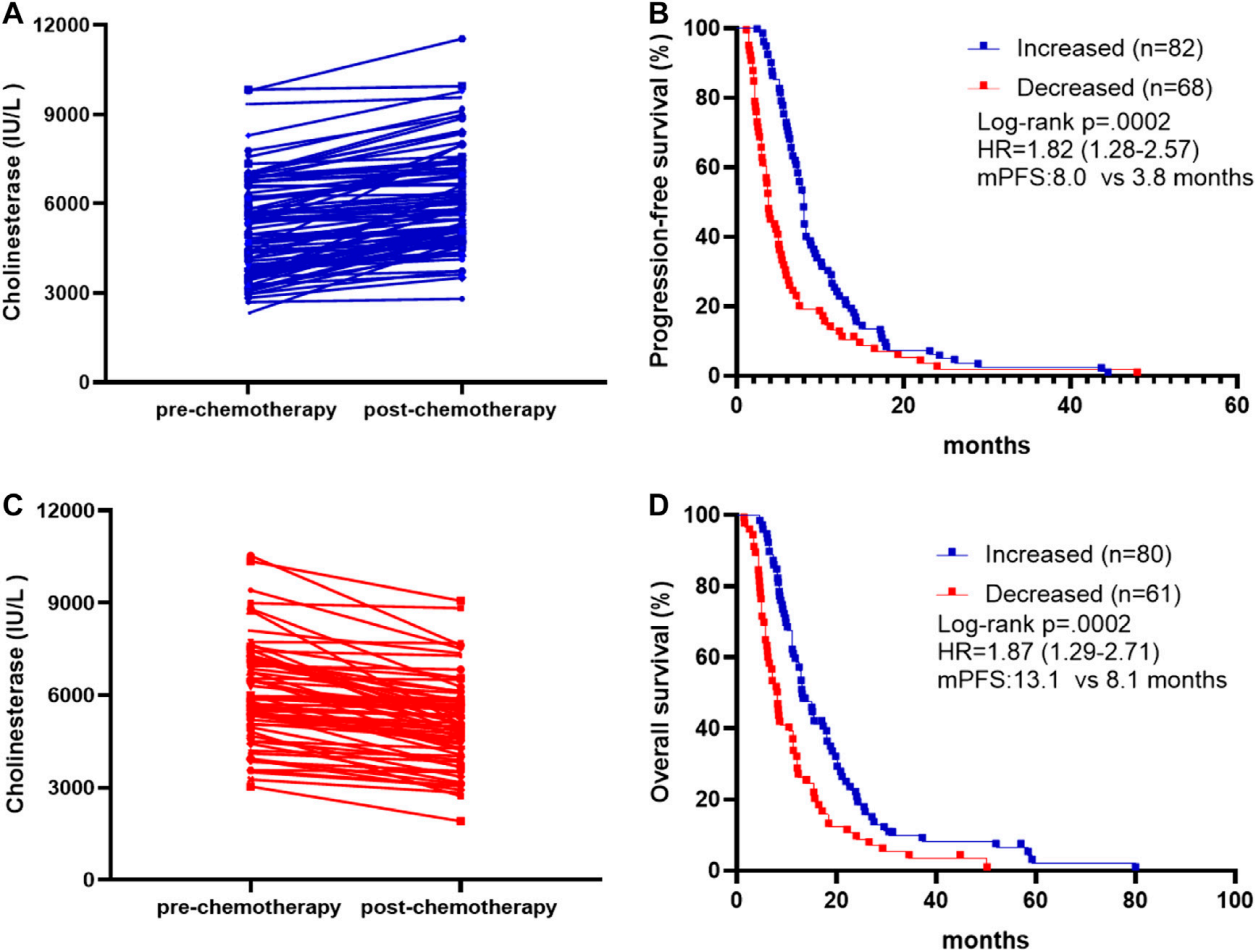
Association between pre-chemotherapy and post-chemotherapy CHE and survival outcomes. **(A)** Patients with increased post-chemotherapy CHE level compared to pre-chemotherapy on. **(B)** Patients with decreased post-chemotherapy CHE level compared to pre-chemotherapy on. **(C)** Kaplan-Meier survival curves of progression-free survival comparing patients with increased and decreased post-chemotherapy CHE. **(D)** Kaplan-Meier survival curves of overall survival comparing patients with increased and decreased post-chemotherapy CHE.

We also investigated whether survival and treatment response depend on the differences in pre- and post-treatment CHE levels, comparing cases with increased, and decreased levels. Post-treatment CHE increased in 82 patients (Figure 2A) and decreased in 68 patients (Figure 2B) than those pre-treatment. The mPFS was 8.0 months in patients with post-treatment CHE increased vs. 3.8 months in patients with CHE decreased after chemotherapy (HR, 1.82; 95% CI 1.28–2.57; *p* = 0.0002; Figure 2C). The mOS was 13.1 months in patients with increased post-treatment CHE vs. 8.1 months in patients with decreased post-treatment CHE (HR, 1.87; 95% CI 1.29–2.71; *p* = 0.0002; Figure 2D).

## Discussion

The identification of markers in GC is important to inform treatment decisions, but also to identify poor prognostic features to inform continuum of care discussions. To our knowledge this is the first report examining the impact of CHE state on treatment outcome in GC. In the present study, we observed that CHE level lower than 5,000 IU/L was associated with poorer PFS, OS and objective response or long-term benefit rate among patients with advanced GC receiving chemotherapy. Multivariate Cox regression analysis confirmed that CHE state was independently and significantly predictive of OS and PFS.

As early as 2005, EAPC guidelines indicated that accurate prediction of survival of patients with advanced tumors is of great importance for palliative care decision making and avoiding overtreatment [11]. Although the ability to diagnose gastric cancer has improved significantly, gastric cancer is still detected at an advanced stage and survival rates for these patients remain poor [4]. Of course, the classification of pathological tumors is valuable in predicting the prognosis of patients with gastric cancer. Many studies have found some independent prognostic factors in tumor-related factors of gastric cancer, such as lymph node status, tumor invasion depth, and molecular markers [12–14]. However, these prognostic factors are not present at the time of gastrectomy or when tissue is not available, as they are largely dependent on histological examination of the specimen. Therefore, it is an important goal to determine a simple and effective prognostic biomarker for patients with advanced gastric cancer before treatment.

Serum CHE activity is often used as an indicator for the diagnosis of organophosphorus poisoning and for the evaluation of the condition after recovery [15]. In clinical practice, it was found that cancer patients were often accompanied by decreased CHE activity [9, 16]. A previous study in TNM stage I-III GC has demonstrated that serum cholinesterase was associated with survival by univariate analysis [5]. However, in this study, we further observed the predictive value of CHE in advanced GC. Indeed, serum level ≥5,000 IU/L is also associated with survival, however, the univariate regression model suggested not only the serum cholinesterase had significant survival difference, but also liver metastasis.

In the present study, we have also demonstrated the potential value of CHE as a real-time marker of chemotherapy effectiveness. In patients with post-treatment CHE increased, the survival rate was significantly higher than patients with CHE decreased after chemotherapy (mPFS: 8.0 vs. 3.8 m, HR, 1.82; 95% CI 1.28–2.57; *p* = 0.0002; mOS: 13.1 vs. 8.1 m, HR, 1.87; 95% CI 1.29–2.71; *p* = 0.0002). These observations open new opportunities for enriching recruitment to studies of novel prognostic indicators in advanced GC with serum level ≥5,000 IU/L. Such novel prognostic indicators could be administered concurrently with imaging diagnosis or alone before and/or after standard chemotherapy is completed. Alternatively, advanced GC with CHE levels above 5,000 IU/L could benefit from more intensive follow-up.

This retrospective study also has several limitations. First, this study lasted from 2013 to 2016. Due to experimental conditions, not all of the patients had HER-2 status detected by IHC. Therefore, HER-2 status was not included in the univariate or multivariate logistic regression analysis of prognosis. Second, we defined 5000 IU/L as the cutoff, while the reference of various hospitals of CHE may vary remarkably and the underlying mechanism between CHE and chemotherapy in GC should be further investigated. But from the existing literature, we found CHE activity in prostate cancer patients is decreased, and its continuous decrease may stimulate the proliferation of prostate cancer cells [8], which may indirectly explain the poor prognosis in patients with CHE levels below 5,000 IU/L. Finally, the sample size of patients with CHE levels below 5,000 IU/L was relatively small, which might restrict the application of the conclusions in the present study, and a larger sample size is needed for study.

In conclusion, this study revealed that advanced GC with CHE levels below 5,000 IU/L was significantly associated with poor PFS and OS. The results suggested that CHE analysis before chemotherapy was a promising prognostic marker for advanced GC.

## Data Availability

The original contributions presented in the study are included in the article/Supplementary Material, further inquiries can be directed to the corresponding author.
